# New structural scaffolds to enhance the metabolic stability of arginine-derived PAD4 inhibitors

**DOI:** 10.1016/j.rechem.2025.102162

**Published:** 2025-02-28

**Authors:** Yijiang Jia, Sina Bahraminejad, Chenyao Jiang, Ayijiang Taledaohan, Dejian Ma, Jianxiong Jiang, Yuji Wang, Jiawang Liu

**Affiliations:** a Medicinal Chemistry Core, Office of Research, University of Tennessee Health Science Center, Memphis, TN 38163, USA.; b Department of Pharmaceutical Sciences, College of Pharmacy, University of Tennessee Health Science Center, Memphis, TN 38163, USA.; c Department of Medicinal Chemistry, School of Pharmaceutical Sciences of Capital Medical University, 10 Xi Tou Tiao, You An Men, Beijing 100069, People’s Republic of China.; d Department of Medicinal Chemistry, Beijing Area Major Laboratory of Peptide and Small Molecular Drugs, Beijing Laboratory of Biomedical Materials, Engineering Research Center of Endogenous Prophylactic of Ministry of Education of China, 10 Xi Tou Tiao, You An Men, Beijing 100069, People’s Republic of China.

**Keywords:** Peptidyl arginine deiminase 4 (PAD4) inhibitor, Citrullination, Metabolic stability, BB-cl-amidine

## Abstract

Although arginine-derived PAD inhibitors represented by Cl-amidine (**2**) showed strong inhibition of PAD4 enzymes and exhibited efficacies in a variety of cellular assays and animal studies, their metabolic instability is a significant challenge for pre-clinical and clinical research. On the basis of the structure of a well-known PAD4 inhibitor BB-Cl-amidine (**3**), we designed two metabolically stable scaffolds, providing two arginine-derived PAD4 inhibitors (**7** and **8**). We evaluated their PAD4 enzyme inhibitory activity *in vitro* and assessed their metabolic stability using liver microsomal assays. These compounds exhibited PAD4 enzyme inhibitory activity (**7**, IC_50_ = 124.93 ± 10.21 μM; **8**, IC_50_ = 46.49 ± 4.46 μM). Hydrolysis of haloacetamidine warheads into hydroxyacetamidine, Compound **7** (t_1/2_ > 60 min), significantly improved the metabolic stability of the lead BB-Cl-amidine (t_1/2_ = 18.11 min). Compound **8** (t_1/2_ > 60 min), the isostere of **7**, also displayed enhanced metabolic stability. Therefore, these two structural scaffolds represent promising new leads for stable PAD4 inhibitors and valuable tools for exploring the reactive cavity of PAD enzymes.

## 1. Introduction

Proteins often undergo various post-translational modifications (PTMs), one of which is citrullination, catalyzed by a group of hydrolases known as protein arginine deiminases (PADs) [1]. This modification affects protein function by converting positively charged arginine residues to neutral citrulline residues [2,3]. Among the PAD family, peptidylarginine deaminase 4 (PAD4) is a calcium-dependent enzyme that is highly expressed in immune cells, such as neutrophils, macrophages, and natural killer (NK) cells, as well as in various malignant tumors [4–6]. Citrullination of nuclear proteins catalyzed by PAD4 disrupts the interaction between positively charged histone residues and negatively charged DNA. This process weakens chromatin structure, resulting in nuclear membrane rupture, chromatin decondensation, and the release of neutrophil extracellular traps (NETs) [6–8]. Furthermore, dysregulation of citrullination has been implicated in a range of diseases, including rheumatoid arthritis (RA), lupus, psoriasis, ulcerative colitis (UC), diabetes, Alzheimer’s disease (AD), inflammatory bowel disease (IBD), and certain cancers [9–15]. Therefore, emerging evidence suggests that PAD4 inhibitors have the potential to treat these diseases [4].

**Fig. 1.**
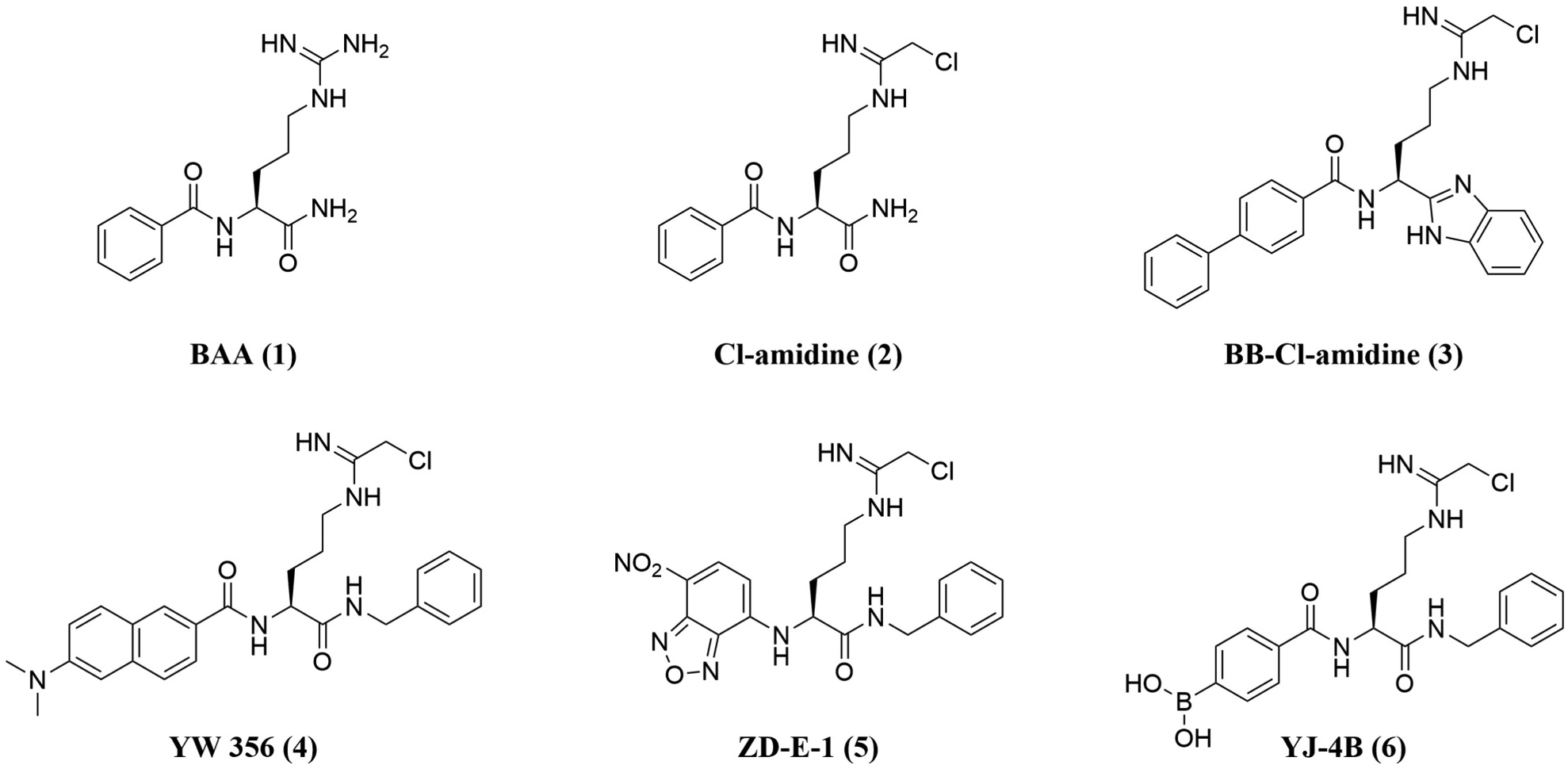
The substrate of PADs (BAA, **1**) and the representatives of PAD4 inhibitors with a Cl-amidine scaffold.

Many covalent and non-covalent inhibitors of PAD4 have been reported recently [5,16,17]. Among these, Cl-amidine (**2**) is a typical irreversible PAD inhibitor that has become a benchmark for evaluating new PAD inhibitors, which incorporates a chloroacetimidamide warhead into the small molecule substrate of PAD enzymes, benzoyl-L-arginine amide (BAA, **1**) [18,19]. Building on Cl-amidine, an analog BB-Cl-amidine (**3**) was developed, featuring a biphenyl group at its N-terminus and a benzimidazole moiety at its C-terminus [6,20]. BB-Cl-amidine retains similar PAD4 inhibitory activity to Cl-amidine *in vitro* while demonstrating significantly enhanced tumor cytotoxicity and improved disease outcomes in an animal model of lupus [20,21]. Despite its extended *in vivo* half-life compared to Cl-amidine, BB-Cl-amidine does not exhibit improved microsomal metabolic stability, which may contribute to its limited efficacy *in vivo* [21,22]. Other irreversible PAD4 inhibitors based on the Cl-amidine scaffold, such as YW-356 (**4**), have also shown rapid metabolism, with a serum circulation time of 5 min [23,24]. Similarly, pharmacokinetic studies revealed short t_1/2_ for ZD-E-1 (**5**) and YJ-4B (**6**), measured at 0.41 h and 0.35 h, respectively [15,24]. (Fig. 1) These findings highlighted the metabolic instability of arginine-derived PAD4 inhibitors, which remains a challenge in advancing them beyond early preclinical development.

**Fig. 2.**
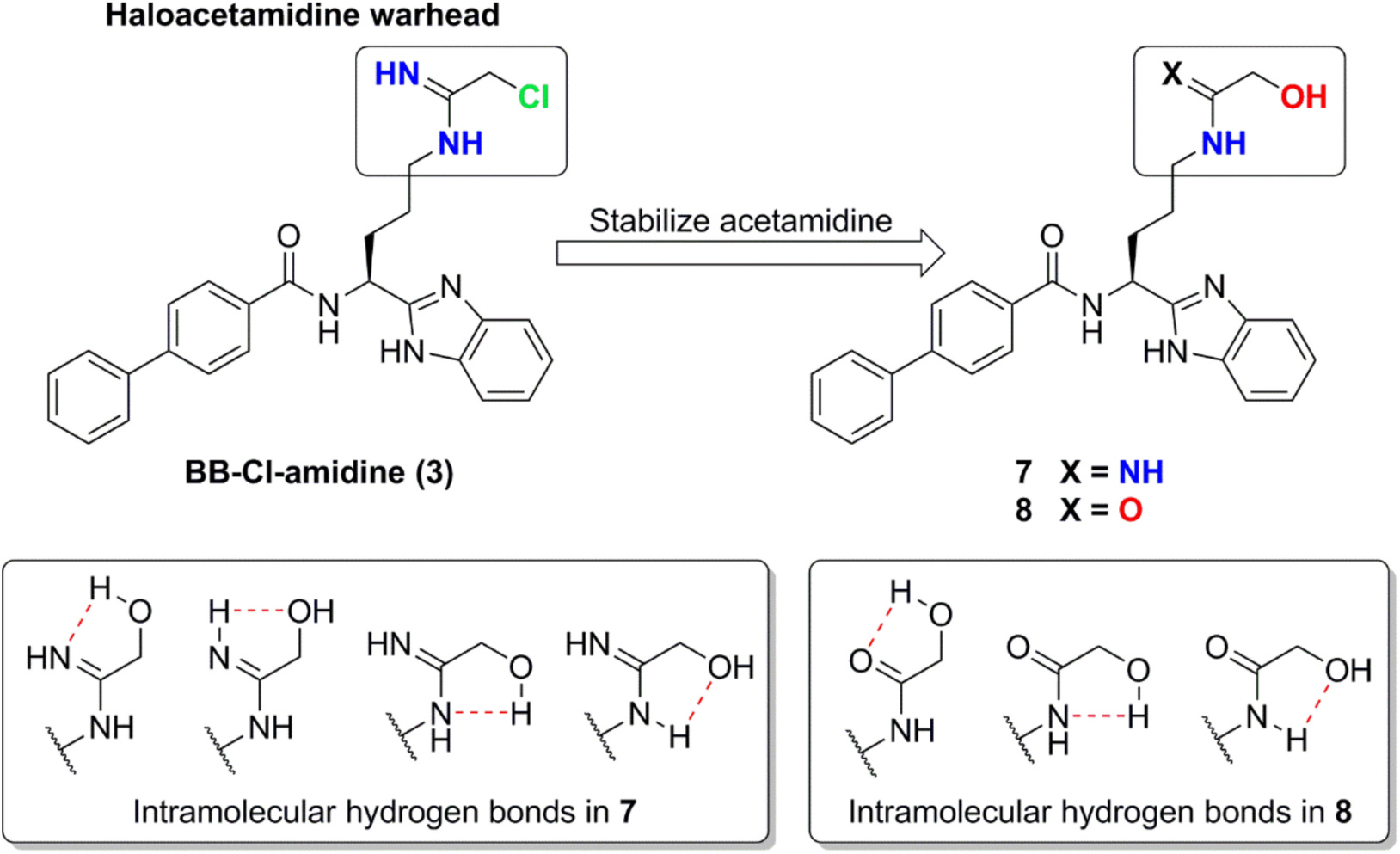
Design of new structural scaffolds for arginine-derived PAD4 inhibitors. Typical irreversible PAD4 inhibitors contain a haloacetamidine warhead, (*e.g*., chlorine or fluorine). In our design, we substituted the halogen with a hydroxyl group to enhance the stability of the imitated arginine residue, allowing it to bind effectively to the PAD4 catalytic cavity. The improved stability of the new scaffolds is partially due to the formation of multiple intramolecular hydrogen bonds enabled by the hydroxyl functional group.

In this study, we designed and synthesized two novel structural scaffolds of arginine-derived PAD4 inhibitors with significantly enhanced metabolic stability (Fig. 2). By replacing the chloroacetimidamide group in BB-Cl-amidine with hydroxyacetimidamide and hydroxyacetamide functional groups, respectively, we developed two new BB-Cl-amidine analogs, **7** and **8**. These analogs demonstrated excellent chemical and biological stability due to the presence of multiple intramolecular hydrogen bonds. Lacking the alphachloride, they avoided nonspecific reactions with proteins or peptides containing thiol groups. Additionally, despite containing hydroxyl groups, the compounds exhibited resistance to phase II drug metabolizing enzymes, a property also attributed to the stabilization of intramolecular hydrogen bonds. The synthesized compounds were evaluated for their PAD4 enzymatic activity and liver microsomal metabolic stability, offering a promising strategy for developing next-generation PAD4 inhibitors with improved oral bioavailability and reduced side effects.

## 2. Results

### 2.1. Synthesis

**Scheme 1.**
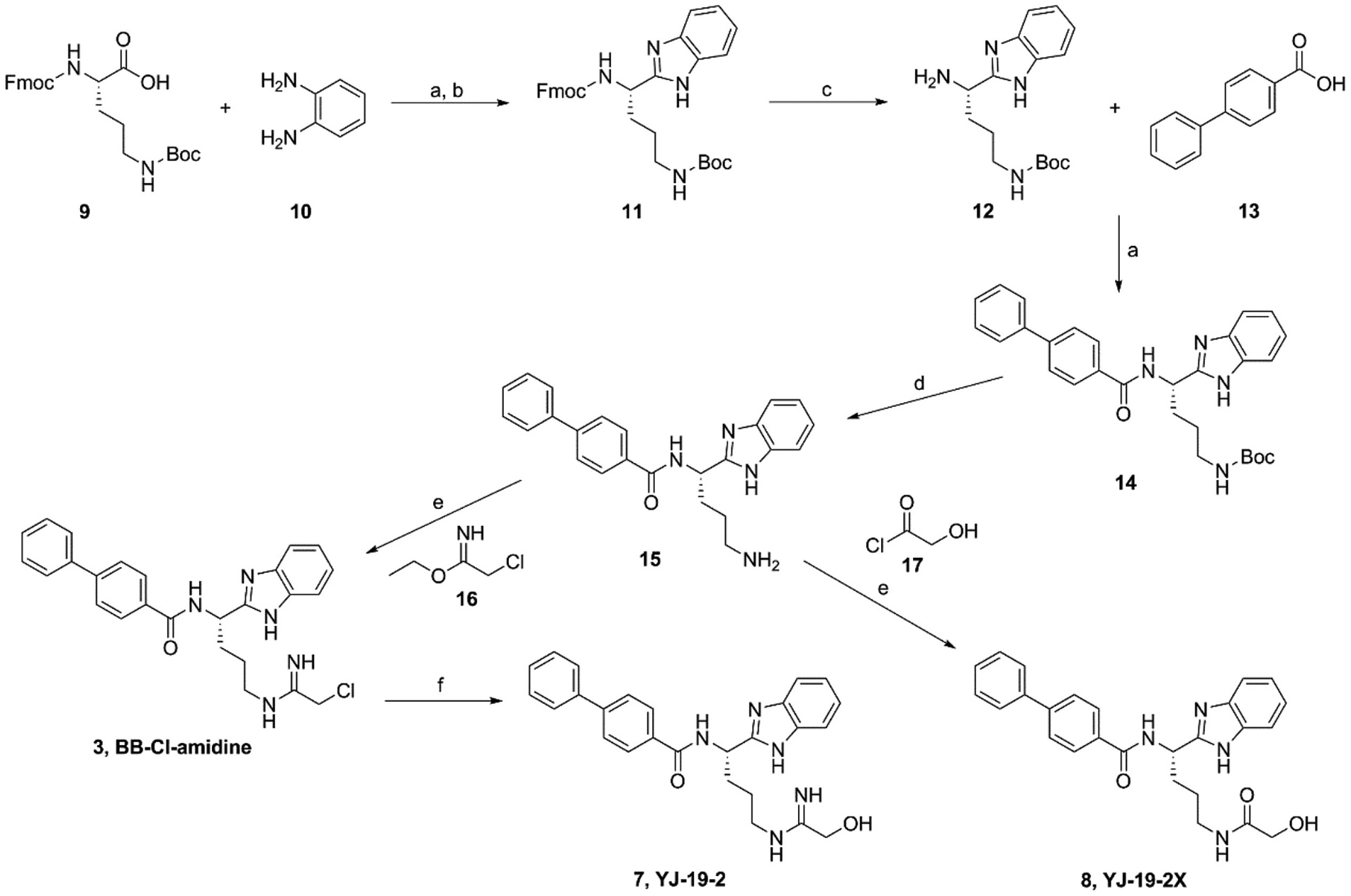
Synthetic route for analogs **7** and **8**. Reagents and conditions: (a) EDC, HOBt, NMM, DMF, 4 h; (b) HAc, 80 °C, 4 h; (c) 20 % piperidine in CH_2_Cl_2_, 3 h; (d) hydrogen chloride, 4 M in 1,4-dioxane, 0 °C, 2 h; (e) anhydrous methanol, DIPEA, 6 h; (f) 5 % NaHCO_3_.

The synthesis of BB-Cl-amidine analogs, **7** and **8**, was depicted in Scheme 1. At room temperature, Fmoc-Orn(Boc)-OH (**9**) and *o*-phenylenediamine (**10**) were coupled with 1-(2-dimethylaminopropyl)-3-ethylcarbodiimide hydrochloride (EDC) in dimethylformamide (DMF) with *N*-methylmorpholine (NMM) as a base to give an amide, which was heated at 80 °C in acetic acid (HAc) to construct the benzoimidazole **11**. After removing the α-NH_2_ protecting group Fmoc of **11** with 20 % piperidine in CH_2_Cl_2_, the amine **12** was coupled with 4-phenylbenzoic acid to form the amide **14**. Then, removing the side chain NH_2_ protecting group of ornithine (Boc), the amine **15** was reacted with ethyl 2-chloroacetimidate in the basic condition to yield BB-Cl-amidine (**3**), which was hydrolyzed in the 5 % NaHCO_3_ to give the analog **7**. HRMS (*m*/*z*): Calcd for C_26_H_28_N_5_O_2_ (M + H)^+^, 442.2238; found, 442.2258. ^1^H NMR (400 MHz, DMSO-*d*_6_) *δ* (ppm) 9.50 (m, 1H), 8.52 (s, 1H), 8.13–8.03 (m, 2H), 7.83–7.67 (m, 5H), 7.55–7.36 (m, 7H), 7.13 (dt, *J* = 5.9, 2.9 Hz, 3H), 5.41 (td, *J* = 8.9, 5.9 Hz, 1H), 4.24 (s, 2H), 3.36–3.27 (m, 2H), 2.19–2.00 (m, 2H), 1.65 (m, 2H). ^13^C NMR (100 MHz, DMSO-*d*_6_) *δ* (ppm) 167.65, 166.39, 155.97, 143.31, 139.61, 133.32, 129.49, 128.81, 128.52, 127.33, 126.84, 121.82, 58.78, 48.78, 48.57, 41.54, 39.60, 31.06, 24.83.

Amine **15** was directly reacted with 2-hydroxyacetyl chloride in a basic condition to give analog **8** with the yield of 84.5 %. HRMS (*m*/*z*): Calcd for C_26_H_27_N_4_O_3_ (M + H)^+^, 443.2078; found, 443.2090. ^1^H NMR (400 MHz, DMSO-*d*_6_) *δ* (ppm) 12.24 (s, 1H), 9.00 (m, 1H), 8.03–7.96 (m, 2H), 7.81–7.63 (m, 5H), 7.43 (t, *J* = 7.5 Hz, 4H), 7.39–7.30 (m, 1H), 7.12–7.02 (m, 2H), 5.40 (s, 1H), 5.27 (td, *J* = 8.8, 5.5 Hz, 1H), 3.71 (s, 2H), 3.11 (q, *J* = 7.1 Hz, 2H), 2.12–1.87 (m, 2H), 1.50 (m, 2H). ^13^C NMR (100 MHz, DMSO-*d*_6_) *δ* (ppm) 172.12, 166.36, 155.93, 143.32, 139.62, 133.32, 129.49, 128.78, 128.53, 127.34, 126.86, 61.90, 48.64, 38.25, 31.21, 26.81.

### 2.2. PAD4 enzyme inhibition assays

To evaluate the PAD4 enzyme inhibitory activities of the three compounds (**3**, **7**, and **8**), we used a PAD4 inhibitor screening kit (Cayman Chemical, Michigan). As shown in Table 1, the positive control BB-Cl-amidine (**3**) exhibited a PAD4 inhibitory activity with an IC_50_ of 1.12 ± 0.06 μM, comparable to Cl-amidine (**2**), as reported in the literature [20]. Phase I drug metabolism processes typically involve oxidation or hydroxylation catalyzed by various enzymes, including cytochrome P450 [25]. For BB-Cl-amidine, we hypothesize that its Cl-amidine component will be rapidly metabolized to OH-amidine or that the amidine group will be hydrolyzed to form an amide *in vivo*. Compound **7**, generated through the hydrolysis of BB-Cl-amidine, showed mild PAD4 inhibition with an IC_50_ of 124.93 ± 10.21 μM. Further modification by replacing the amidine group of **7** with an amide group produced **8**, which demonstrated improved PAD4 inhibitory activity with an IC_50_ of 46.49 ± 4.46 μM.

Research based on crystal structures and biochemical studies suggests that the catalysis of the PAD4 enzyme follows a reverse protonation process [26,27]. The enzymatic mechanism is initiated as the thiolate of Cys645 nucleophilically attacks the guanidine carbon of the substrate, forming a covalent tetrahedral intermediate [28]. Thus, irreversible PAD inhibitors, such as Cl-amidine or BB-Cl-amidine, covalently modify the cysteine residues at the active site, eventually inactivating the enzyme [21,28]. Because the modification of cysteine requires an electron-withdrawing effect (such as Cl or F) on the α-position of the imine group to facilitate the reaction between Cys645 of the enzyme and the imine carbon of inhibitors, hydrolysis of the chloroacetimidamide group (BB-Cl-amidine, **3**) to a hydroxyacetimidamide or a hydroxyacetamide group (analog **7** or **8**) could avoid this covalent binding [28,29]. Therefore, we hypothesized that analogs **7** and **8** act as non-covalent (or reversible) PAD4 inhibitors.

**Fig. 3.**
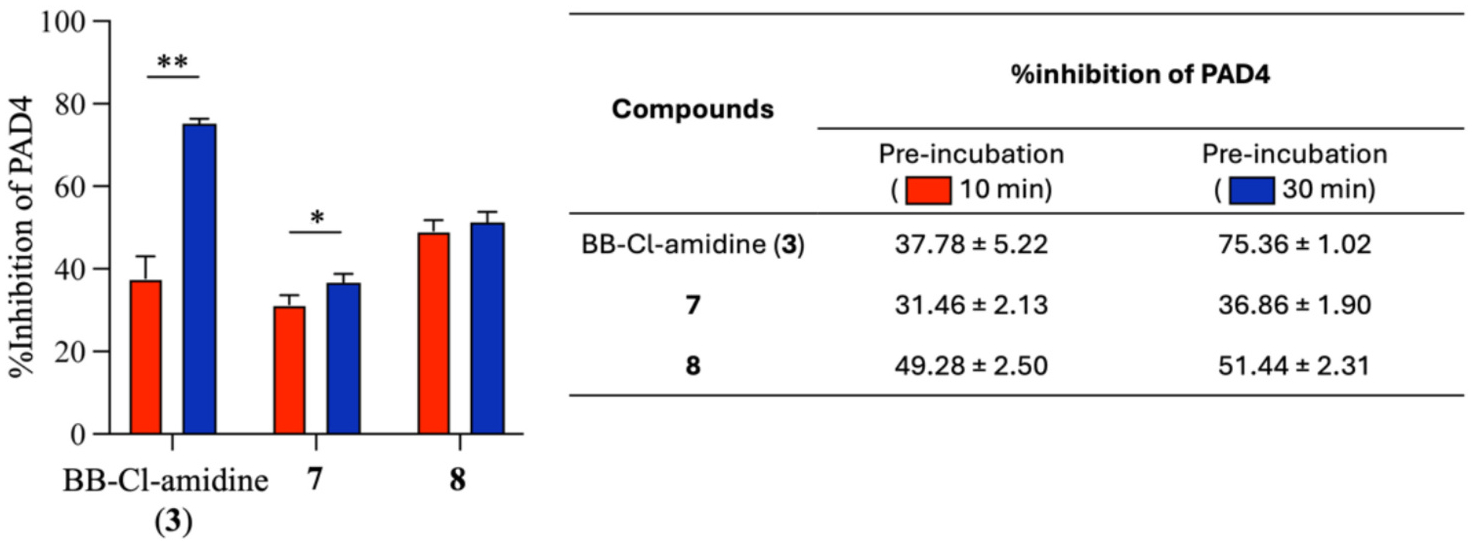
Inhibition of PAD4 with a pre-incubation of the enzyme and tested compounds for 10- or 30-min. The final concentrations of BB-Cl-amidine, **7**, and **8** were 1 μM, 125 μM, and 45 μM, respectively. The inhibition of PAD4 by BB-Cl-amidine (**3**) showed obvious time-dependent characteristics, while the inhibitory effects of **7** and **8** were either unaffected or only slightly influenced by incubation time. *, *p* < 0.05. **, *p* < 0.01. *n* = 3.

To test this hypothesis, the three compounds (**3**, **7** and **8**) were further assessed through a time-dependent inhibition assay. This assay involved pre-incubating the compounds with the PAD4 enzyme for 10 and 30 min before initiation of the enzymatic reaction. BB-Cl-amidine (**3**) demonstrated time-dependent inhibition, with the percentage of inhibition increasing from 37.78 % to 75.36 %, as shown in Fig. 3. In contrast, the inhibitory activities of **7** (from 31.46 % to 36.86 %) and **8** (from 49.28 % to 51.44 %) exhibited minimal changes over time. These results suggest that **7** and **8** are reversible PAD4 inhibitors that interact with the enzyme through non-covalent binding.

The PAD1 and PAD2 inhibitory activities of the three compounds (BB-Cl-amidine, **7**, and **8**) were determined as shown in Table 1. Consistent with literature reports, BB-Cl-amidine exhibits significantly stronger inhibitory activity against PAD1 compared to other isozymes [20]. The inhibitory activity of Compound **7** against PAD4 (IC_50_ = 124.93 ± 10.21 μM) is superior to its activity against PAD1 (IC_50_ = 194.27 ± 8.21 μM) and PAD2 (IC_50_ = 352.10 ± 21.30 μM), suggesting it is a potential selective PAD4 inhibitor. In contrast, although Compound **8** exhibits relatively stronger overall inhibitory activity against the PAD family compared to Compound **7**, its inhibitory activity against PAD1 (IC_50_ = 31.98 ± 0.78 μM) is more potent than that against PAD4 (IC_50_ = 46.49 ± 4.46 μM).

The cytotoxicity of three compounds on A549 cells was evaluated using the MTT assay. At concentrations of 50 μM and 5 μM, BB-Cl-amidine, Compound **7**, and Compound **8** showed no cytotoxicity. As shown in Fig. S1, at a test concentration of 500 μM, the cell viability treated with BB-Cl-amidine was 60.45 %, while that of Compound **7** was reduced to 26.76 %, and Compound **8** remained at 102.73 %. The IC_50_ values of BB-Cl-amidine and **8** against A549 cells exceeded 500 μM. Further determination revealed that the IC_50_ value of Compound **7** was estimated to be 454.7 μM. These results indicate that BB-Cl-amidine, **7**, and **8** all exhibit low cytotoxicity and demonstrate good cellular safety within the experimental concentration range, providing evidence for the safety profile of arginine-derived PAD inhibitors.

### 2.3. Microsomal metabolic stability assays

**Fig. 4.**
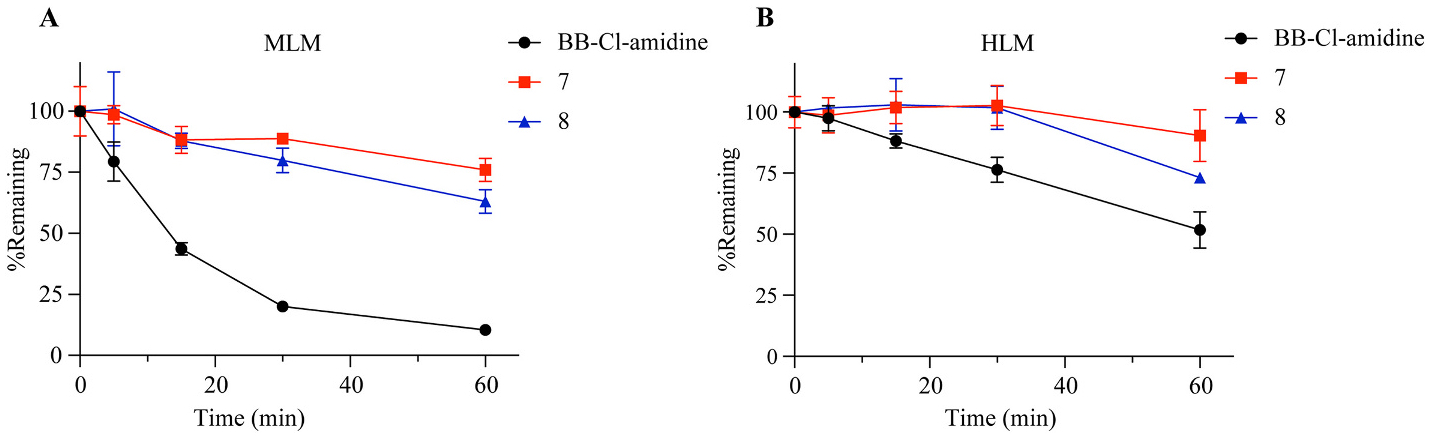
Remaining curves of compounds **3**, **7**, and **8** in mouse liver and human liver microsomal metabolism. (*n* = 3).

The metabolic stability of the compounds was evaluated using mouse liver microsomes (MLM) and human liver microsomes (HLM). Each compound was tested at a final concentration of 0.2 μM to determine their half-lives. As shown in Fig. 4, the half-life (t_1/2_) of BB-Cl-amidine was 18.11 min in murine liver microsomes and 61.94 min in human liver microsomes. In contrast, the metabolic stability of compounds 7 and 8 was significantly enhanced in both murine and human liver microsomes (**7**: t_1/2_ > 60 min in MLM and HLM; **8**: t_1/2_ > 60 min in MLM and HLM). These findings suggest that **7** and **8** exhibit promising stability and potential for oral administration in future animal studies.

To further highlight the differences in microsomal stability among the tested compounds, the percentage of remaining compound after 60 min is shown in Table 2. At an initial concentration of 0.2 μM, the remaining of BB-Cl-amidine in MLM was 10.45 %, whereas compounds **7** and **8** demonstrated significantly higher values of 75.92 % and 63.02 %, respectively. Rough estimations of the t_1/2_ values for **7** and **8** in MLM were approximately 159 and 87 min, respectively. A similar trend was observed in HLM, with remaining percentages of 51.79 % for BB-Cl-amidine, 90.33 % for **7**, and 73.15 % for **8**. These results indicate that compounds **7** and **8** are considerably more metabolically stable than BB-Cl-amidine, with **7** exhibiting the highest stability.

## 3. Conclusions

In summary, starting from the lead compound BB-Cl-amidine, we developed two structural scaffolds that retain the arginine-like side chain essential for effective binding to the PAD4 enzyme. At the same time, these scaffolds eliminate the reactive chloroacetimidamide group, thereby improving metabolic stability and reducing non-specific interactions with proteins or peptides containing thiol residues. The two representative compounds, **7** and **8**, exhibited mild and reversible inhibition of PAD4 *in vitro*, while demonstrating significantly improved metabolic stability in mouse and human liver microsomes compared to the lead compound, BB-Cl-amidine. The enhanced stability is expected to contribute to improved pharmacokinetic properties and pharmacological effects. Based on these findings, we predict that the new structural scaffolds will enable mild and sustained inhibition of PAD4 *in vivo*. Future structural modifications according to these two scaffolds could pave the way for developing orally available small-molecule PAD4 inhibitors.

## Supplementary Material

Supplementary material

## Figures and Tables

**Table 1:** *In vitro* PADs Inhibition by BB-Cl-amidine, **7**, and **8**.

Compounds	Structures	Inhibitory activity IC50 (μM)		
		PAD4	PAD2	PAD1
3, BB-cl-amidine	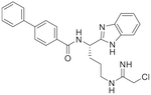	1.12 ± 0.06	3.38 ± 0.27	0.53 ± 0.09
7	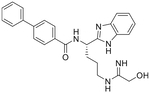	124.93 ± 10.21	352.10 ± 21.30	194.27 ± 8.21
8	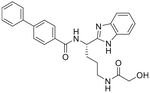	46.49 ± 4.46	74.93 ± 6.48	31.98 ± 0.78

(*n* = 3)

**Table 2:** 60 min %remaining of compounds in liver microsomal metabolism assays.

Compounds	Concentration (μM)	%remaining in MLM	%remaining in HLM
BB-cl-amidine (3)	0.2	10.45 ± 1.42	51.79 ± 7.48
7	0.2	75.92 ± 4.75	90.33 ± 10.64
8	0.2	63.02 ± 4.85	73.15 ± 1.89

(n = 3)

## Data Availability

Data will be made available on request.
