# Supplementary material for: New structural scaffolds to enhance the metabolic stability of arginine-derived PAD4 inhibitors
Source: Results Chem. Author manuscript; Available in PMC 2025 May 29. (PMC12121987; doi:10.1016/j.rechem.2025.102162)
Supplement: Supplementary material [file NIHMS2083528-supplement-Supplementary_material.pdf]

Supporting Information

New Structural Scaffolds to Enhance the Metabolic Stability of Arginine-Derived PAD4 Inhibitors

Yijiang Jia[a,b], Sina Bahraminejad[b], Chenyao Jiang[b], Ayijiang Taledaohan[c,d], Dejian Ma[b], Jianxiong Jiang[b], Yuji Wang[c,d,*], Jiawang Liu[a,b,*]

a. Medicinal Chemistry Core, Office of Research, University of Tennessee Health Science Center, Memphis, TN 38163, USA.

b. Department of Pharmaceutical Sciences, College of Pharmacy, University of Tennessee Health Science Center, Memphis, TN 38163, USA.

c. Department of Medicinal Chemistry, School of Pharmaceutical Sciences of Capital Medical University, 10 Xi Tou Tiao, You An Men, Beijing 100069, People's Republic of China.

d. Department of Medicinal Chemistry, Beijing Area Major Laboratory of Peptide and Small Molecular Drugs, Beijing Laboratory of Biomedical Materials, Engineering Research Center of Endogenous Prophylactic of Ministry of Education of China, 10 Xi Tou Tiao, You An Men, Beijing 100069, People's Republic of China.

*Corresponding author. Tel./fax: +86 10 83911530.

E-mail address: wangyuji@ccmu.edu.cn (Yuji Wang).

*Corresponding author. Tel./fax: +(901)448-2372.

E-mail address: jliu90@uthsc.edu (Jiawang Liu).

Contents

Materials 3
Synthesis 3
PAD4 Enzyme Inhibition Assays 7
Microsomal metabolic stability assay protocol 7
MTT assay 9
Figure S1 Cell viability(%) of A549 cells 9
Figure S2 The mass spectrum Compound 14 10
Figure S3 HRMS spectrum of Compound 7 11
Figure S4 HRMS spectrum of Compound 8 12
Figure S5 ^{1}H NMR (400 MHz, DMSO-*d6*) spectra of Compound 14 13
Figure S6 ^{13}C NMR (100 MHz, DMSO-*d6*) spectra of Compound 14 14
Figure S7 ^{1}H NMR (400 MHz, DMSO-*d6*) spectra of Compound 7 15
Figure S8 ^{13}C NMR (100 MHz, DMSO-*d6*) spectra of Compound 7 16
Figure S9 ^{1}H NMR (400 MHz, DMSO-*d6*) spectra of Compound 8 17
Figure S10 ^{13}C NMR (100 MHz, DMSO-*d6*) spectra of Compound 8 18

Experimental Section

Materials

Fmoc-Orn(Boc)-OH and o-phenylenediamine were purchased from TCI AMERICA; 4-Biphenylcarboxylic Acid, Ethyl 2-Chloroacetimidate Hydrochloride, Hydroxyacetyl chloride, and BB-Cl-amidine were purchased from AmBeed; HOBt and EDC were purchased from Enamine; N,N-dimethylformamide (DMF), methanol, dichloromethane, hexanes, and ethyl acetate were purchased from Fisher chemical; Dioxane hydrochloride, acetonitrile for chromatography, and formic acid for chromatography were purchased from Thermo Scientific; N,N-diisopropylethylamine (DIPEA), NMM, diethylamine were purchased from Alfa Aesar.

Synthesis

*Synthesis of **11***. Fmoc-Orn(Boc)-OH (0.909 g, 2 mmol, 1.0 eq) was dissolved in 5 mL of anhydrous DMF. Under an ice bath, 0.297 g of HOBt (2.2 mmol, 1.1 eq) and 0.420 g of EDC·HCl (2 mmol, 1.0 eq) were added and completely dissolved. While still under the ice bath, 0.238 g of o-phenylenediamine (2 mmol, 1.1 eq) was added to the reaction mixture and the pH was adjusted to 8-9 using N-methylmorpholine (NMM). After stirring at room temperature for 4 h, TLC (hexanes: ethyl acetate = 1:1) showed the disappearance of Fmoc-Orn(Boc)-OH. The reaction mixture was diluted with 150 mL of ethyl acetate and sequentially washed three times each with saturated $NaHCO_3$ solution, saturated NaCl solution, 5% $KHSO_4$ solution, saturated NaCl solution, saturated $NaHCO_3$ solution, and saturated NaCl solution. The ethyl acetate layer was dried over anhydrous $MgSO_4$, and the filtrate was concentrated under reduced pressure to dryness. The residue was washed three times with hexanes to obtain intermediate in 78.1% yield. ESI-MS (m/z): 545.27 $[M+H]^+$ (Exact Mass: 544.27, $C_{31}H_{36}N_4O_5$). Then, the intermediate (850 mg) was dissolved in 20 mL of glacial acetic acid. The reaction mixture was heated at 80°C for 4 h. TLC (hexanes: ethyl acetate = 1:1) showed the disappearance of intermediate. Upon completion of the reaction, the acetic acid was evaporated to dryness and the residue was redissolved in 150 mL of ethyl acetate. The resulting solution was washed sequentially three times

each with saturated $NaHCO_3$ solution, saturated NaCl solution, 5% $KHSO_4$ solution, saturated NaCl solution, saturated $NaHCO_3$ solution and saturated NaCl solution. The ethyl acetate layer was dried over anhydrous $MgSO_4$ and the filtrate was concentrated under reduced pressure to dryness, yielding compound **11** with a yield of 79.3%. ESI-MS (m/z): 527.32 $[M+H]^+$ (Exact Mass: 526.27, $C_{31}H_{34}N_4O_4$).

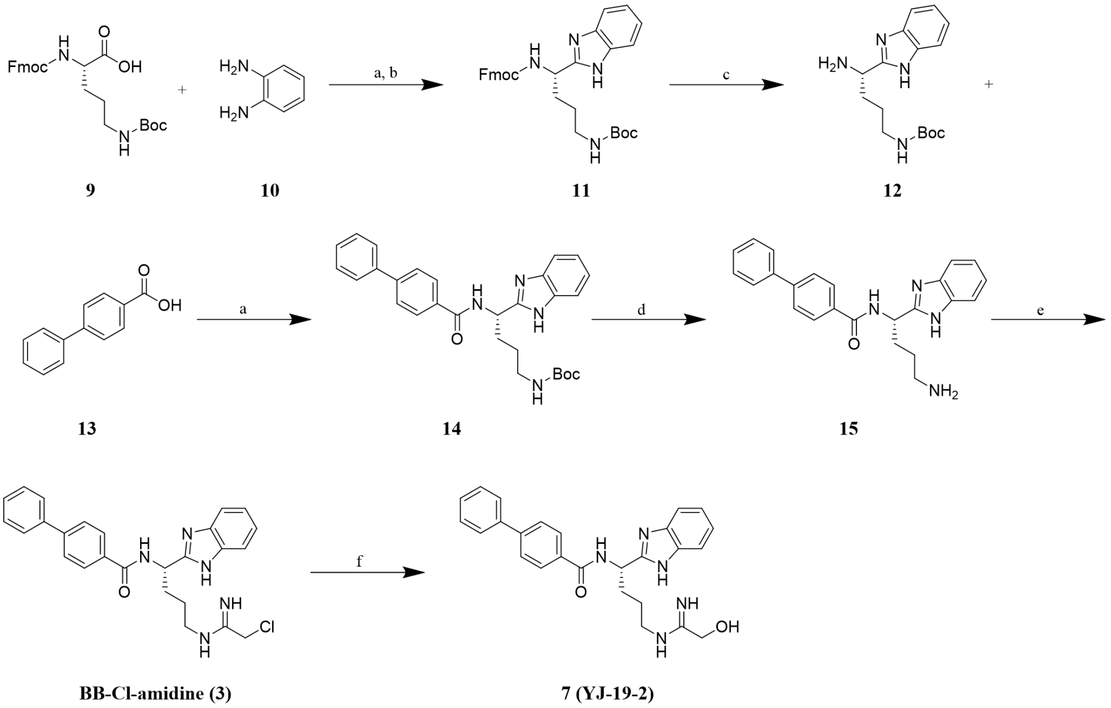

Scheme 1 Synthesis of **7** (YJ-19-2)

Reagents and conditions: (a) EDC, HOBt, NMM, DMF, 4 h; (b) HAc, 80 °C, 4 h; (c) 20% piperidine in CH_2Cl_2, 3 h; (d) hydrogen chloride, 4M in 1,4-dioxane, 0°C, 2 h; (e) anhydrous methanol, DIPEA, 6 h; (f) 5% $NaHCO_3$.

*Synthesis of **12***. Compound **11** (650 mg) was dissolved in 50 mL of CH_2Cl_2 containing 20% diethylamine. The reaction mixture was stirred at room temperature for 3 h. TLC (dichloromethane:methanol = 20:1) showed the disappearance of Compound **11**. After evaporating the solvent, the product was triturated with petroleum ether to disperse the product uniformly in hexanes. The hexanes were then removed to obtain compound **12** in 93.3% yield. ESI-MS (m/z): 305.20 $[M+H]^+$ (Exact Mass:304.19, $C_{16}H_{24}N_4O_2$).

*Synthesis of **14***. Compound **14** was synthesized using a similar synthetic method as that of intermediate for compound **11**. Compound **14** was obtained in 86.1% yield using 4-phenylbenzoic acid (118 mg, 0.6 mmol, 1.0 eq) as a raw material. ESI-MS (m/z): 485.26 $[M+H]^+$ (Exact Mass: 484.25, $C_{29}H_{32}N_4O_3$). ^{1}H-NMR (400 MHz, DMSO-d_6) δ (ppm) 12.27 (s, 1H), 8.07 (dd, J = 8.6, 2.7 Hz, 2H), 7.77 (dd, J = 22.3, 7.7 Hz, 4H), 7.66 (s, 1H), 7.57 (d, J = 7.4 Hz, 1H), 7.56 – 7.37 (m, 4H), 7.15 (q, J = 6.6 Hz, 2H), 6.87 (t, J = 5.5 Hz, 1H), 5.33 (td, J = 8.7, 5.5 Hz, 1H), 2.99 (q, J = 6.7 Hz, 2H), 2.17 – 1.97 (m, 2H), 1.59 – 1.43 (m, 2H), 1.36 (s, 9H). ^{13}C NMR (100 MHz, DMSO-d_6) δ (ppm) 166.37, 156.01, 155.92, 143.44, 143.32, 139.62, 134.61, 133.34, 129.49, 128.78, 128.53, 127.34, 126.85, 122.24, 121.47, 118.88, 111.69, 77.83, 48.57, 31.17, 28.72, 26.87.

*Synthesis of **15***. Dissolve compound **14** (160 mg) in 2 mL of 1,4-dioxane. Then 1.5 mL of dioxane hydrochloride was added in an ice bath. The reaction mixture was stirred at room temperature for 2 h. TLC (dichloromethane: methanol = 20:1) showed that compound **14** disappeared. The product was washed with hexanes to obtain compound **15** in 99.9% yield. ESI-MS (m/z): 385.20 $[M+H]^+$ (Exact Mass:384.20, $C_{24}H_{24}N_4O$).

Synthesis of BB-Cl-amidine (3). Compound **15** (100 mg, 0.26 mmol, 1.0 eq) was dissolved in an appropriate amount of anhydrous methanol and stirred. Under an ice-water bath, ethyl 2-chloroacetimidate hydrochloride (205 mg, 1.3 mmol, 5.0 eq) was added and the pH was adjusted to 10 using N,N-diisopropylethylamine (DIPEA). The reaction mixture was stirred at room temperature for 6 h. TLC (ethyl acetate:water:acetic acid = 40:10:1) showed the disappearance of Compound **15**. The solvent was evaporated under reduced pressure to dryness. The mixture was purified by C18 column chromatography with ACN:H_2O (0.02% formic acid) as the mobile phase, and then lyophilized to obtain BB-Cl-amidine (**3**) in 63.9% yield. ESI-MS (m/z): 460.21 $[M+H]^+$ (Exact Mass:459.18, $C_{26}H_{26}ClN_5O$).

Synthesis of YJ-19-2 (7). Dissolve 75 mg of BB-Cl-amidine in 3 mL of 5% $NaHCO_3$ solution and react until BB-Cl-amidine disappears by LC-MS. After purification by C18 column chromatography using ACN:H_2O (0.02% formic acid) as mobile phase, YJ-19-2 (**7**) was lyophilized in 86.3% yield. HRMS (m/z): Calcd for $C_{26}H_{28}N_5O_2$ $(M+H)^+$, 442.2238; found, 442.2258. ^{1}H-NMR (400 MHz, DMSO-d_6) δ (ppm) 9.50 (m, 1H), 8.52 (s, 1H), 8.13 – 8.03 (m, 2H), 7.83 – 7.67 (m, 5H), 7.55 – 7.36 (m, 7H), 7.13 (dt, J = 5.9, 2.9 Hz, 3H), 5.41 (td, J = 8.9, 5.9 Hz, 1H), 4.24 (s, 2H), 3.36 – 3.27 (m, 2H), 2.19 – 2.00 (m, 2H), 1.65 (m, 2H). ^{13}C NMR (100 MHz, DMSO-d_6) δ (ppm) 167.65, 166.39, 155.97, 143.31, 139.61, 133.32, 129.49, 128.81, 128.52, 127.33, 126.84, 121.82, 58.78, 48.78, 48.57, 41.54, 39.60, 31.06, 24.83.

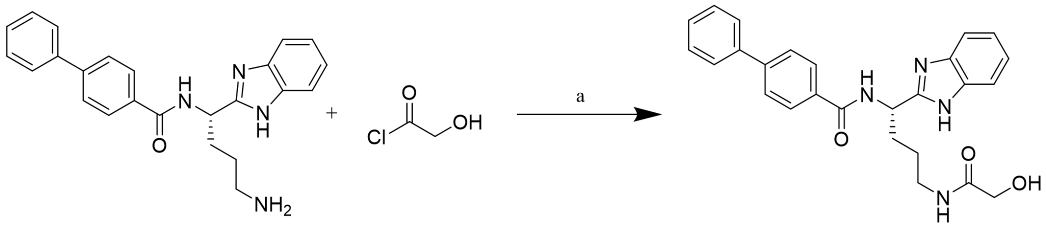

15 **17** **8 (YJ-19-2X)**

Scheme 2 Synthesis of **8** (YJ-19-2X)

Reagents and conditions: (a) Anhydrous methanol, DIPEA, RT, 6 h.

*Synthesis of YJ-19-2X (**8**)*. Compound **15** (35 mg, 0.09 mmol, 1.0 eq) was dissolved in an appropriate amount of anhydrous methanol and stirred. Under an ice-water bath, 2-hydroxyacetyl chloride (25.8 mg, 0.27 mmol, 3.0 eq) was added and the pH was adjusted to 10 using N,N-diisopropylethylamine (DIPEA). The reaction mixture was stirred at room temperature for 6 h. TLC (ethyl acetate:water:acetic acid = 40:10:1) showed the disappearance of Compound **15**. The solvent was evaporated under reduced pressure to dryness. The mixture was purified by C18 column

chromatography with ACN:H_2O (0.02% formic acid) as the mobile phase, and then lyophilized to obtain YJ-19-2X (**8**) in 84.8% yield. HRMS (m/z): Calcd for $C_{26}H_{27}N_4O_3$ $(M+H)^+$, 443.2078; found, 443.2090. ^1H NMR (400 MHz, DMSO-d_6) δ (ppm) 12.24 (s, 1H), 9.00 (m, 1H), 8.03 – 7.96 (m, 2H), 7.81 – 7.63 (m, 5H), 7.43 (t, J = 7.5 Hz, 4H), 7.39 – 7.30 (m, 1H), 7.12 – 7.02 (m, 2H), 5.40 (s, 1H), 5.27 (td, J = 8.8, 5.5 Hz, 1H), 3.71 (s, 2H), 3.11 (q, J = 7.1 Hz, 2H), 2.12 – 1.87 (m, 2H), 1.50 (m, 2H). ^{13}C NMR (100 MHz, DMSO-d_6) δ (ppm) 172.12, 166.36, 155.93, 143.32, 139.62, 133.32, 129.49, 128.78, 128.53, 127.34, 126.86, 61.90, 48.64, 38.25, 31.21, 26.81.

PAD4 Enzyme Inhibition Assays

PAD4 Inhibitor Screening Assay Kit (AMC) was purchased from Cayman. Different concentrations of the test compounds were added to a 96-well plate with PAD4 enzyme enzyme and incubated at 37 °C for 10 minutes or 30 minutes. Then 7-amino-4-methylcoumarin (AMC) was added to initiate the reaction and incubated at 37 °C for 20 min. The reaction was terminated by the addition of Develop solution. Fluorescence analysis was performed at excitation wavelengths of 355 ~ 365 nm and emission wavelengths of 445 ~ 455 nm. The fluorescence signal was inversely proportional to the amount of citrullination. IC_{50} were analyzed using GraphPad Prism 10.3.0.

Microsomal metabolic stability assay protocol

Pooled liver microsomes from male C57BL/6 mouse (MLM) and mixed gender human (HLM) were obtained from Sekisui XenoTech (Kansas City, KS). Stock solutions of compounds (1 mM) were prepared in DMSO and diluted to 10 μM working solution using phosphate buffer (100 mM, pH 7.4). Microsomes (20 mg/mL) were thawed on ice and diluted to 1 mg/mL using potassium phosphate buffer (100 mM, pH 7.4). The mixture of liver microsomes and test compounds (0.2 μM) was

preincubated at 37°C for 10 min. Next, NADPH-regenerating system (final concentrations of 1 mM $NADP^+$, 5 mM glucose-6-phosphate, and 1 U/mL glucose-6-phosphate dehydrogenase) as a cofactor was added to the incubation mixtures at the final volume of 1000 µL to initiate the metabolic reactions. Quenching of the metabolic reaction was performed at specific intervals: 0, 5, 15, 30, and 60 min by adding100 µL of reaction mixtures to ice-cold acetonitrile (4 volumes) containing 25 nM internal standard. The samples were kept on ice for 15 minutes, followed by protein precipitation through centrifugation at 3600 rpm for 10 minutes at 4°C. Finally, the liquid chromatography-mass spectrometry/MS (LC-MS/MS) analysis was conducted on obtained supernatants. To ensure accuracy, all measurements were conducted in triplicate.

The percent of remaining compound (%R) compared to zero sample time was obtained at each time point using LC-MS peak areas. A plot was created for the natural logarithm (ln) of % R against the incubation time to determine the elimination rate constant from the slope of the linear part. The *in vitro* $t_{1/2}$ was measured by following Eq. (1):

$$t1/2 = \frac{Ln2}{Slope}$$

While the following Eq. (2) was used for calculating CLint (µL/min/mg):

$$CLint = \frac{Ln2}{in\ vitro\ t1/2} \times \frac{Volume\ of\ incubation\ (\mu L)}{Amount\ of\ microsomal\ protein\ (mg)}$$

MTT assay

In vitro cytotoxicity was evaluated using the MTT (3-(4,5-dimethylthiazol-2-yl)-2,5-diphenyltetrazolium bromide) assay. Freshly trypsinized A549 cells were seeded into 96-well plates at a density of 5×10^4 cells/mL, with 100 µL per well, and incubated at 37°C in a 5% CO_2 atmosphere for 12 hours. Subsequently, BB-Cl-amidine, **7**, and **8** were added to the 96-well plate at final concentrations of 500 µM, 50 µM, and 5 µM (25 µL per well), followed by 48-hour incubation. After this period, MTT solution (5 mg/mL) was added to each well at 25 µL per well, and the plates were incubated for an additional 4 hours. Control wells containing untreated cells were handled identically. Following incubation, the medium in the 96-well plate was removed, and 100 µL of DMSO was added to each well to dissolve the formazan crystals produced by the reduction of MTT in viable cells. Absorbance was measured using a microplate reader. Cell viability was determined using the following formula: Cell viability (%) = (OD-treated cells)/(OD control cells) × 100%.

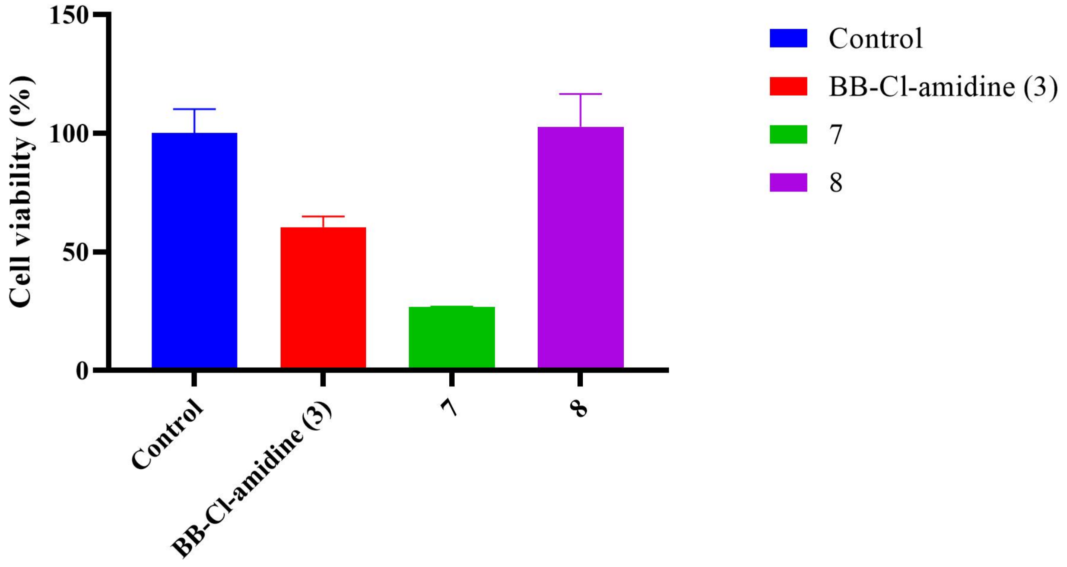

Figure S1 Cell viability(%) of A549 cells at 500 µM concentration of the three compounds.

Display Report

Analysis Info

Analysis Name	D:\Data\Yijiang Jia\20240903-484-5_1-44_01_14529.d	Acquisition Date	9/3/2024 10:18:44 AM
Method	5_min_lcms_method set_14529.m	Operator	bruker
Sample Name	20240903-484-5	Instrument	amaZon SL
Comment			

Acquisition Parameter

Ion Source Type	ESI	Ion Polarity	Positive	Alternating Ion Polarity	off
Mass Range Mode	UltraScan	Scan Begin	50 m/z	Scan End	1500 m/z
Accumulation Time	200000 µs	RF Level	63 %	Trap Drive	53.1
SPS Target Mass	400 m/z	Averages	5 Spectra	n/a	n/a

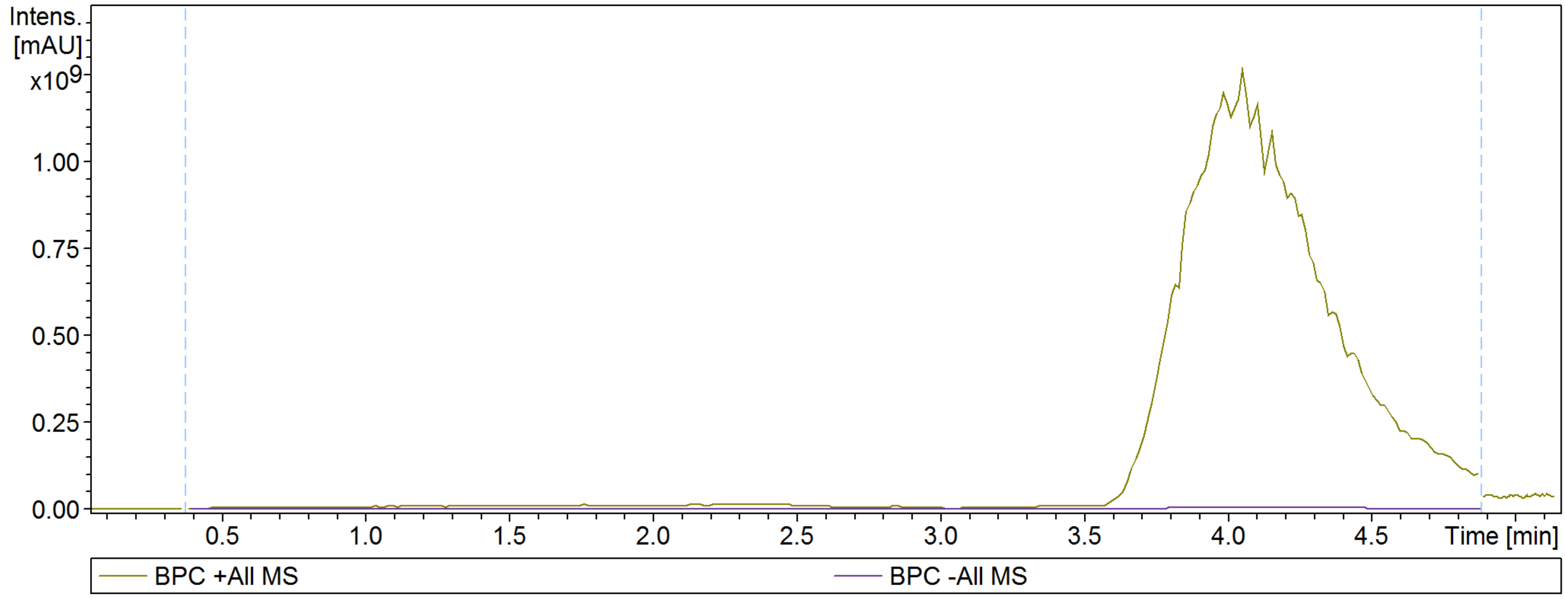

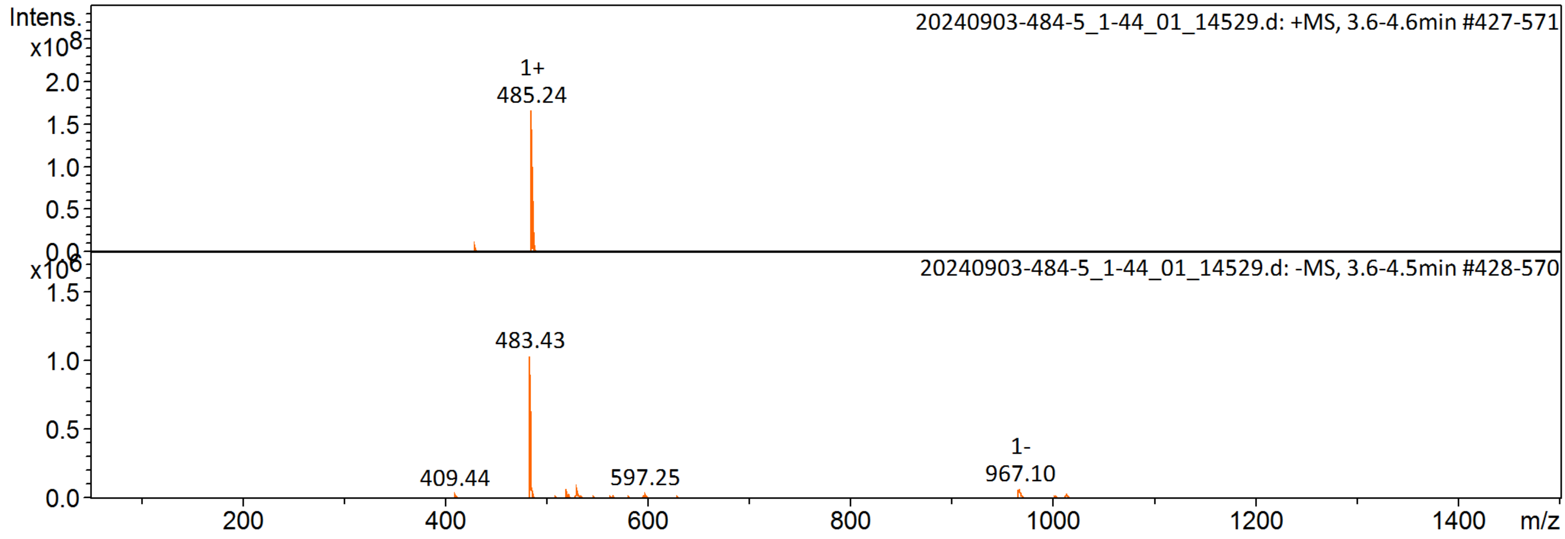

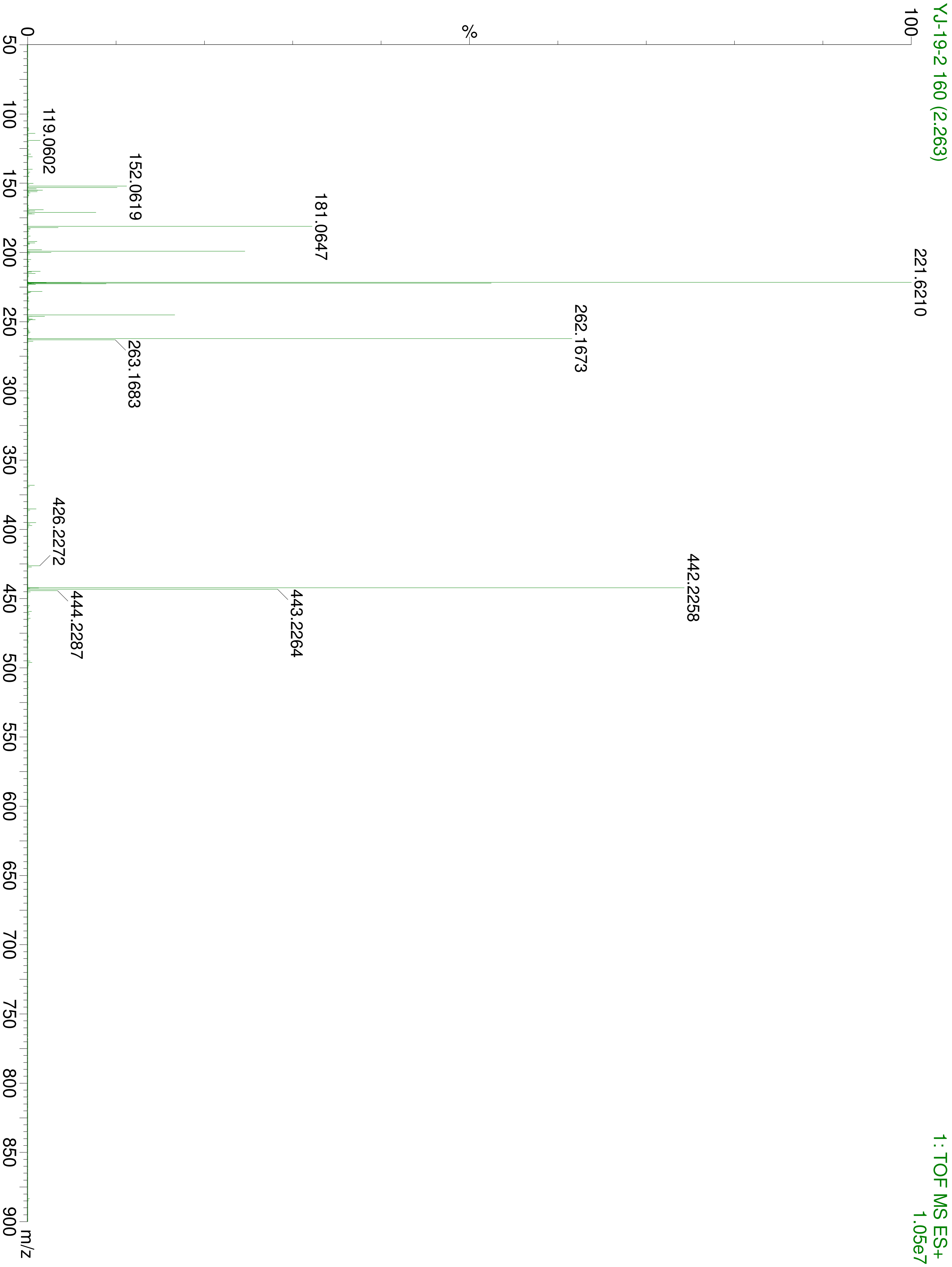
YJ-19-2 160 (2.263)
1: TOF MS ES+
1.05e7
119.0602
152.0619
181.0647
221.6210
262.1673
263.1683
426.2272
442.2258
443.2264
444.2287
%
m/z

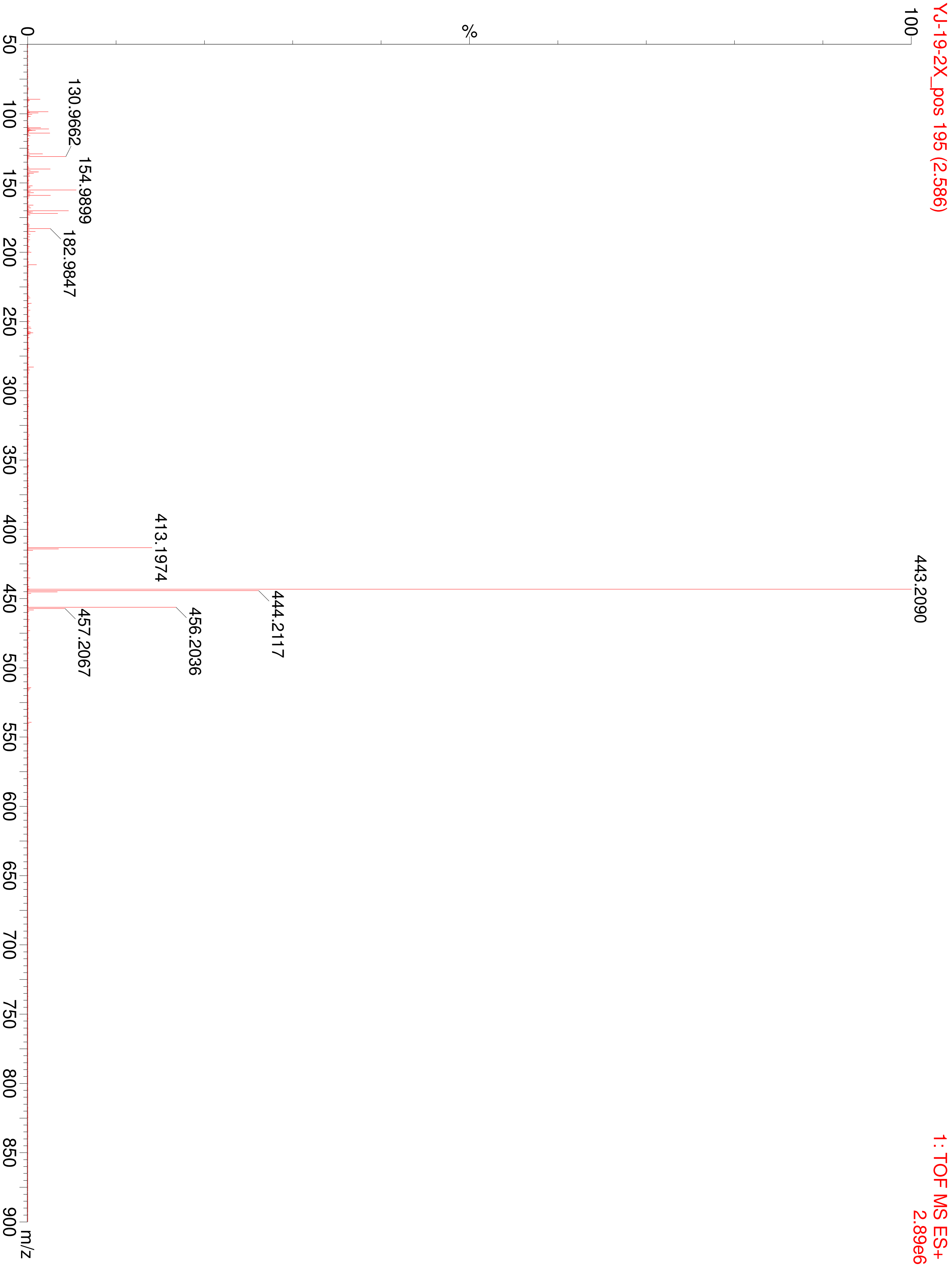
YJ-19-2X_pos 195 (2.586)
1: TOF MS ES+
2.89e6
100
%
0
443.2090
444.2117
456.2036
457.2067
413.1974
130.9662
154.9899
182.9847
50
100
150
200
250
300
350
400
450
500
550
600
650
700
750
800
850
900
m/z

14 (^{1}H NMR)

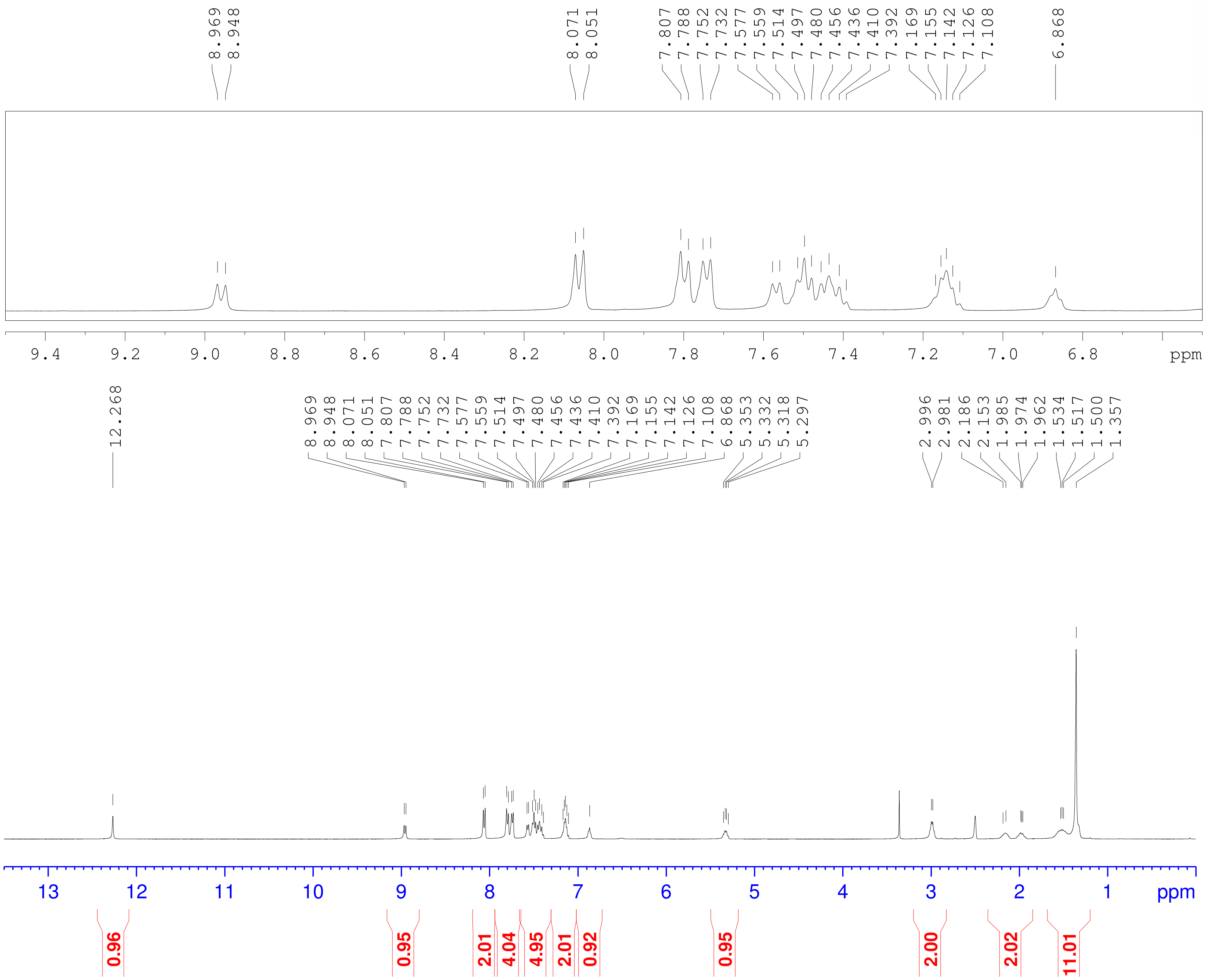

THE UNIVERSITY OF TENNESSEE HEALTH SCIENCE CENTER
Office of Research
Medicinal Chemistry Core
881 Madison Ave, Room 579
Memphis, TN 38163

```
Current Data Parameters
NAME      20240906-BB-Orn(Boc)-Mizuo
EXPNO                      1
PROCNO                     1

F2 - Acquisition Parameters
Date_               20240906
Time                   11.23 h
INSTRUM                spect
PROBHD   Z108618_0396 (
PULPROG                 zg30
TD                     65536
SOLVENT                 DMSO
NS                        16
DS                         2
SWH                 8012.820 Hz
FIDRES              0.244532 Hz
AQ                 4.0894465 sec
RG                     119.4
DW                    62.400 usec
DE                     16.92 usec
TE                     293.2 K
D1               1.00000000 sec
TD0                        1
SFO1             400.1324708 MHz
NUC1                      1H
P0                      5.00 usec
P1                     15.00 usec
PLW1             11.40600014 W

F2 - Processing parameters
SI                     65536
SF               400.1299973 MHz
WDW                       EM
SSB       0
LB                      0.30 Hz
GB        0
PC                      1.00
```

14 (^{13}C NMR)

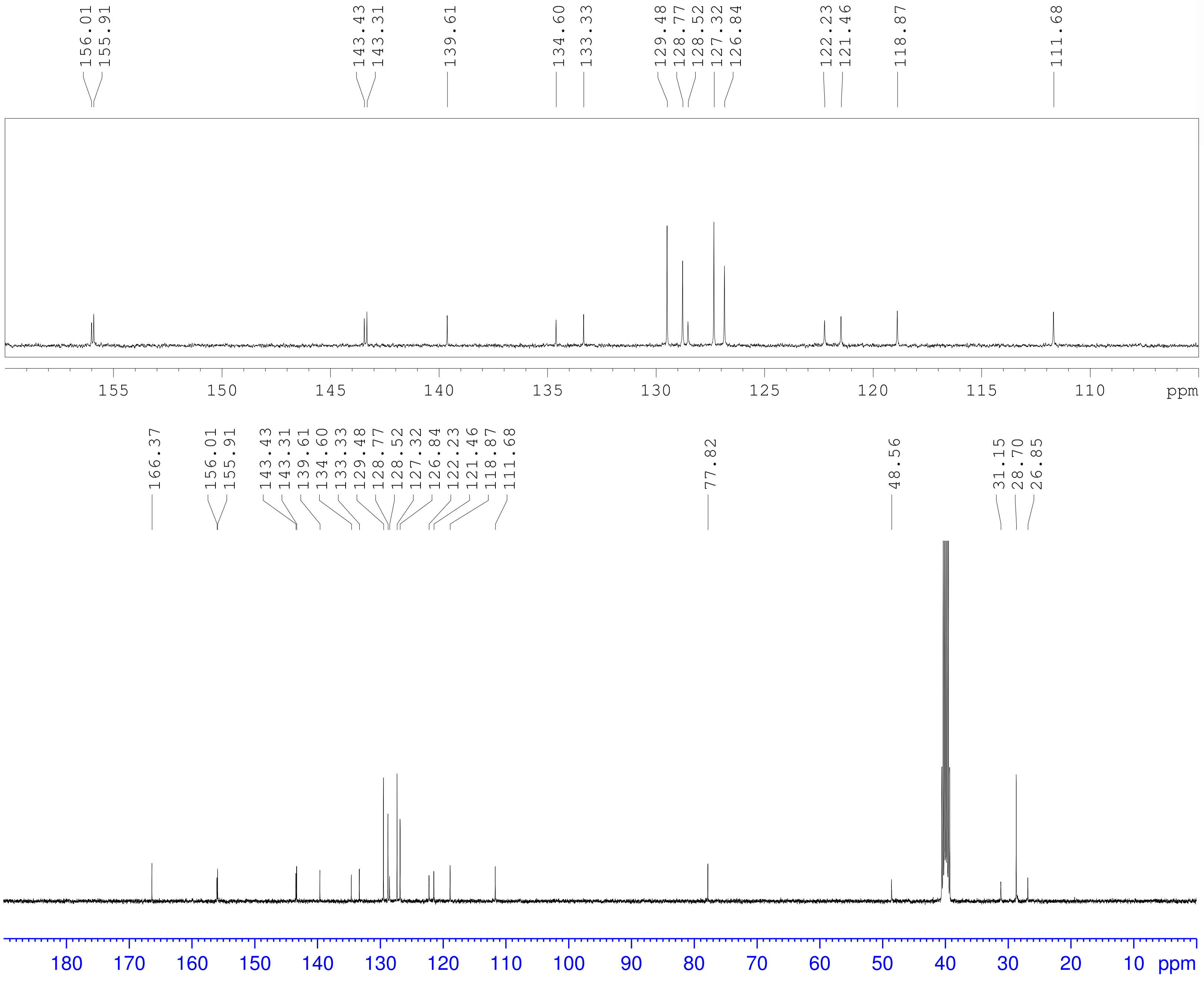

THE UNIVERSITY OF TENNESSEE HEALTH SCIENCE CENTER.

Office of Research
Medicinal Chemistry Core
881 Madison Ave, Room 579
Memphis, TN 38163

Current Data Parameters

NAME	20240906-BB-Orn(Boc)-Mizuo	
EXPNO	2	
PROCNO	1	

F2 - Acquisition Parameters

Date_	20240906	
Time	21.32	h
INSTRUM	spect	
PROBHD	Z108618_0396 (	
PULPROG	zgpg30	
TD	65536	
SOLVENT	DMSO	
NS	1024	
DS	4	
SWH	24038.461	Hz
FIDRES	0.733596	Hz
AQ	1.3631488	sec
RG	199.69	
DW	20.800	usec
DE	6.50	usec
TE	293.2	K
D1	2.00000000	sec
D11	0.03000000	sec
TD0	1	
SFO1	100.6228298	MHz
NUC1	13C	
P0	3.08	usec
P1	9.25	usec
PLW1	56.00000000	W
SFO2	400.1316005	MHz
NUC2	1H	
CPDPRG[2	waltz65	
PCPD2	90.00	usec
PLW2	15.00000000	W
PLW12	0.36296001	W
PLW13	0.18257000	W

F2 - Processing parameters

SI	32768	
SF	100.6127709	MHz
WDW	EM	
SSB	0	
LB	1.00	Hz
GB	0	
PC	1.40	

7, YJ-19-2 (^{1}H NMR)

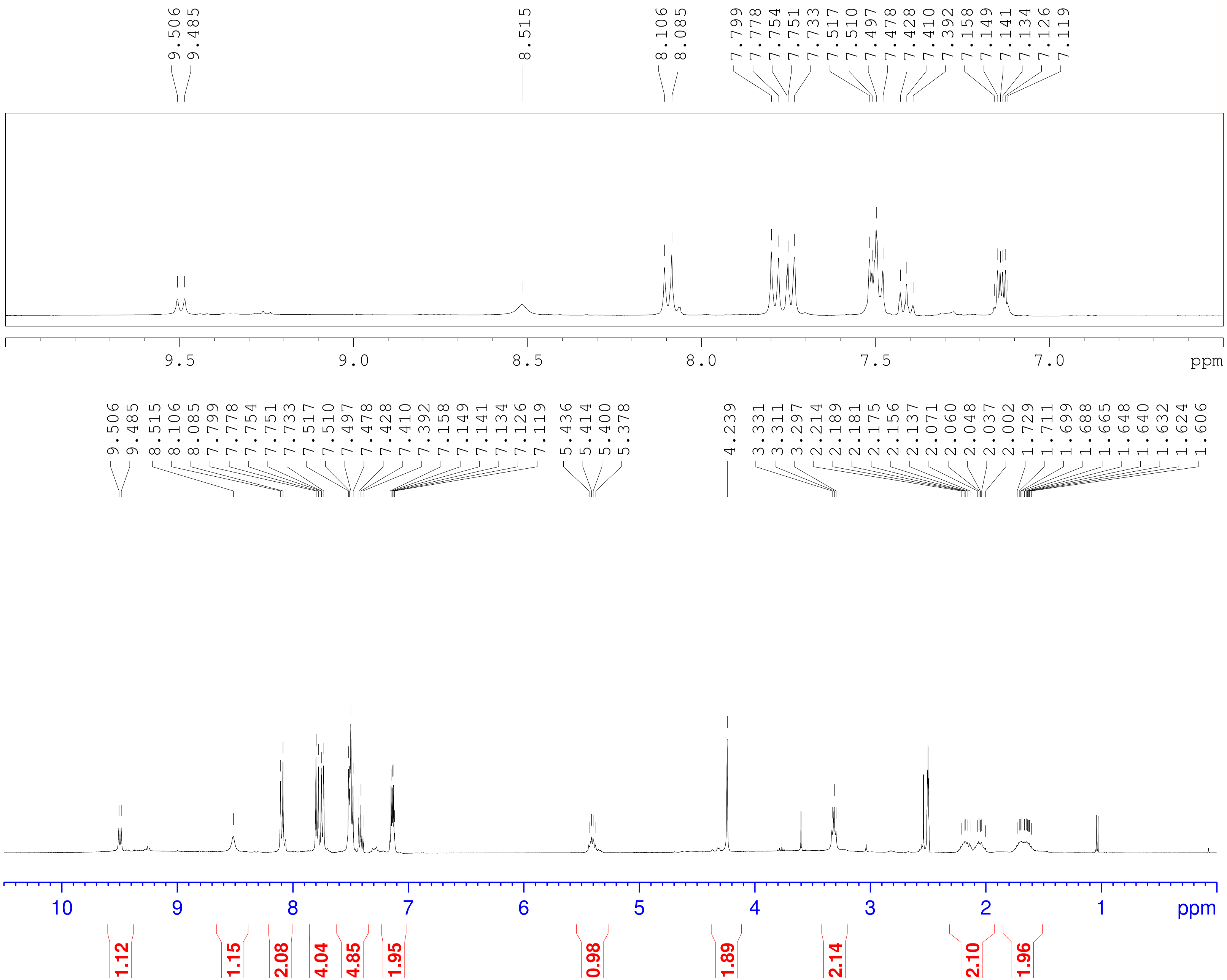

THE UNIVERSITY OF TENNESSEE HEALTH SCIENCE CENTER

Office of Research
Medicinal Chemistry Core
881 Madison Ave, Room 579
Memphis, TN 38163

Current Data Parameters
NAME 20241115-YJ-19-2
EXPNO 1
PROCNO 1

F2 - Acquisition Parameters
Date_ 20241115
Time 13.09 h
INSTRUM spect
PROBHD Z108618_0396 (
PULPROG zg30
TD 65536
SOLVENT DMSO
NS 16
DS 2
SWH 8012.820 Hz
FIDRES 0.244532 Hz
AQ 4.0894465 sec
RG 132.71
DW 62.400 usec
DE 16.92 usec
TE 293.2 K
D1 1.00000000 sec
TD0 1
SFO1 400.1324708 MHz
NUC1 1H
P0 5.00 usec
P1 15.00 usec
PLW1 11.40600014 W

F2 - Processing parameters
SI 65536
SF 400.1299963 MHz
WDW EM
SSB 0
LB 0.30 Hz
GB 0
PC 1.00

7, YJ-19-2 (^{13}C NMR)

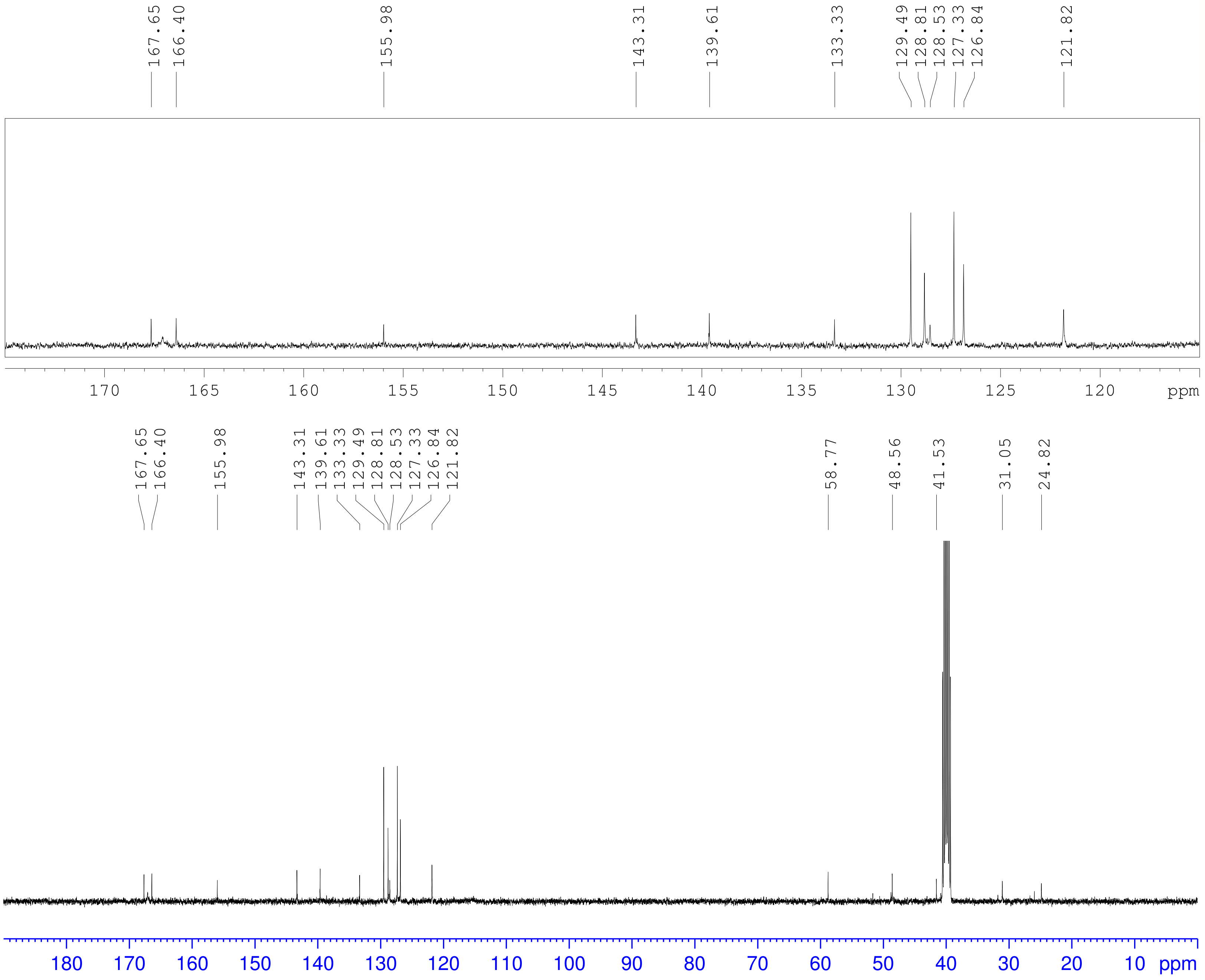

THE UNIVERSITY OF TENNESSEE HEALTH SCIENCE CENTER.

Office of Research
Medicinal Chemistry Core
881 Madison Ave, Room 579
Memphis, TN 38163


```
Current Data Parameters
NAME     20241115-YJ-19-2
EXPNO                   2
PROCNO                  1

F2 - Acquisition Parameters
Date_            20241115
Time                23.08 h
INSTRUM             spect
PROBHD     Z108618_0396 (
PULPROG            zgpg30
TD                  65536
SOLVENT              DMSO
NS                   1024
DS                      4
SWH             24038.461 Hz
FIDRES           0.733596 Hz
AQ              1.3631488 sec
RG                 199.69
DW                 20.800 usec
DE                   6.50 usec
TE                  293.2 K
D1             2.00000000 sec
D11            0.03000000 sec
TD0                     1
SFO1          100.6228298 MHz
NUC1                  13C
P0                   3.08 usec
P1                   9.25 usec
PLW1          56.00000000 W
SFO2          400.1316005 MHz
NUC2                   1H
CPDPRG[2          waltz65
PCPD2               90.00 usec
PLW2          15.00000000 W
PLW12          0.36296001 W
PLW13          0.18257000 W

F2 - Processing parameters
SI                  32768
SF            100.6127698 MHz
WDW                    EM
SSB      0
LB                   1.00 Hz
GB       0
PC                   1.40
```

8, YJ-19-2X (^{1}H NMR)

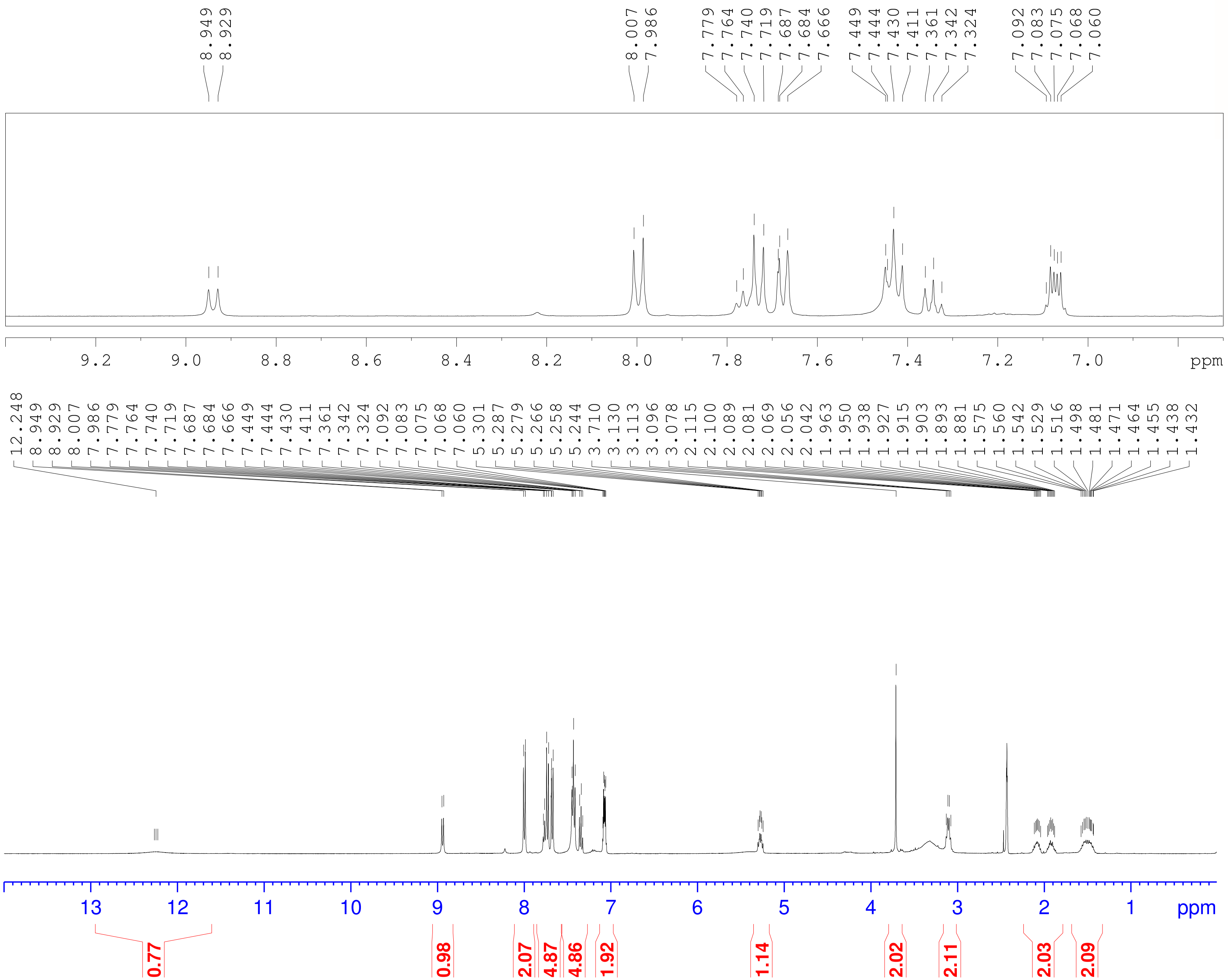

THE UNIVERSITY OF TENNESSEE
HEALTH SCIENCE CENTER

Office of Research
Medicinal Chemistry Core
881 Madison Ave, Room 579
Memphis, TN 38163

```
Current Data Parameters
NAME      20241115-YJ-19-2X
EXPNO                     1
PROCNO                    1

F2 - Acquisition Parameters
Date_              20241115
Time                  13.12 h
INSTRUM               spect
PROBHD   Z108618_0396 (
PULPROG                zg30
TD                    65536
SOLVENT                DMSO
NS                       16
DS                        2
SWH                8012.820 Hz
FIDRES             0.244532 Hz
AQ                4.0894465 sec
RG                   159.26
DW                   62.400 usec
DE                    16.92 usec
TE                    293.2 K
D1               1.00000000 sec
TD0                       1
SFO1            400.1324708 MHz
NUC1                     1H
P0                     5.00 usec
P1                    15.00 usec
PLW1            11.40600014 W

F2 - Processing parameters
SI                    65536
SF              400.1300246 MHz
WDW                      EM
SSB      0
LB                     0.30 Hz
GB       0
PC                     1.00
```

8, YJ-19-2X (^{13}C NMR)

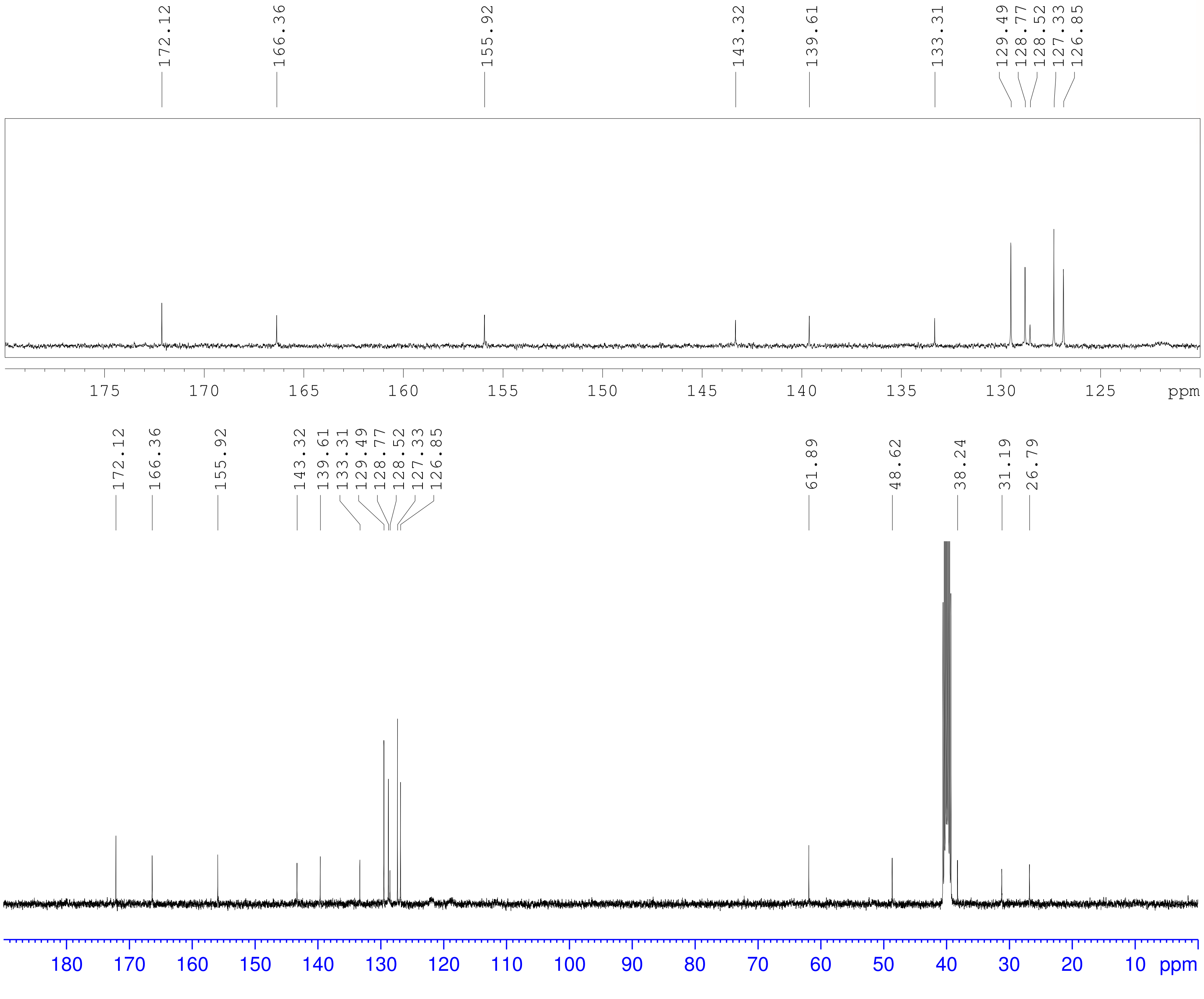

THE UNIVERSITY OF TENNESSEE HEALTH SCIENCE CENTER.

Office of Research
Medicinal Chemistry Core
881 Madison Ave, Room 579
Memphis, TN 38163

Current Data Parameters

NAME	20241115-YJ-19-2X
EXPNO	2
PROCNO	1

F2 - Acquisition Parameters

Date_	20241116
Time	0.09 h
INSTRUM	spect
PROBHD	Z108618_0396 (
PULPROG	zgpg30
TD	65536
SOLVENT	DMSO
NS	1024
DS	4
SWH	24038.461 Hz
FIDRES	0.733596 Hz
AQ	1.3631488 sec
RG	199.69
DW	20.800 usec
DE	6.50 usec
TE	293.2 K
D1	2.00000000 sec
D11	0.03000000 sec
TD0	1
SFO1	100.6228298 MHz
NUC1	13C
P0	3.08 usec
P1	9.25 usec
PLW1	56.00000000 W
SFO2	400.1316005 MHz
NUC2	1H
CPDPRG[2	waltz65
PCPD2	90.00 usec
PLW2	15.00000000 W
PLW12	0.36296001 W
PLW13	0.18257000 W

F2 - Processing parameters

SI	32768
SF	100.6127707 MHz
WDW	EM
SSB	0
LB	1.00 Hz
GB	0
PC	1.40
